# Book Reviews

**Published:** 1892-03

**Authors:** 


### Book Reviews.

TEXT BOOK OF MEDICAL JURISPRUDENCE AND TOXICOLOGY.—By John J. Reese, M. D., Professor of Medical Jurisprudence and Toxicology, in University of Pennsylvania. P. Blakiston, Son & Co., Philadelphia.

The third edition of this most excellent text book is now out. As a clear, concise and well arranged text book, we think it is one of the best before the profession. The new edition contains fifty-five more pages than the former a few subjects having been somewhat enlarged.

In Chapter VI. a new section has been added, and under the head of death by poisoning, a new division has been made into, first, classes and a sub-division, then into orders and arrangement, we think advantageous. In the appendix the former subject of *corpus luteum* of pregnancy has been omitted, and that of protracted voluntary fasting added.

Dr. Reese gives a brief history of the celebrated Signor Giovanni Succi, in New York, and gives some brief remarks touching upon this subject.

Every physician should have a good standard work on Medical Jurisprudence in his library, and I know of none better to be recommended than this work of Dr. Reese.

C. D. R.

---

W. B. SAUNDERS has now in preparation, and will have ready for delivery about June 1st, 1892, "An American Text Book of Surgery," by Professors Keen, White, Burnett, Conner, Dennis, Park, Nancrede, Pilcher, Senn, Shepherd, Stimson, Thomson and Warren, forming one volume of about 1,200 pages, handsomely illustrated and bound in cloth and sheep. Price of former, $7.00; latter, $8.00.

Also, "An American Text Book of the Theory and Practice of Medicine, according to American Teachers," edited by William Pepper, M. D., L L. D., to be completed in two handsome volumes, of about 1,000 pages each, illustrated. Price per volume, cloth, $5.00; sheep, $6.00; half Russia, $7.00.

